# Cytomorphological Spectrum of Thyroiditis: A Review of 110 Cases

**DOI:** 10.1155/2018/5246516

**Published:** 2018-03-01

**Authors:** Shirish S. Chandanwale, Rahul Nair, Anushree Gambhir, Supreet Kaur, Aditi Pandey, Abhinav Shetty, Piyusha Naragude

**Affiliations:** ^1^Department of Pathology, Dr. D. Y. Patil Medical College, Pimpri, Pune 411018, India; ^2^Dr. D. Y. Patil Medical College, Pimpri, Pune 411018, India

## Abstract

**Introduction:**

Different types of thyroiditis may share some parallel clinical and biochemical features. Timely intervention can significantly reduce morbidity and mortality.

**Aim:**

Aim of this study is to find the frequency of various thyroiditis, study the cytomorphological features and correlate with clinical findings including radiological findings, thyroid function test, and anti-thyroid peroxidase antibodies (Anti-TPO antibodies).

**Materials and Methods:**

The study included consecutive 110 cases of thyroiditis. Detailed cytomorphological features were studied and correlated with ultrasonography findings, thyroid function test, anti-thyroid peroxidase antibodies (anti-TPO) and histopathological features where thyroidectomy specimens were received for histopathological examination.

**Results:**

The majority were Hashimoto's thyroiditis (*n* = 100) and females (*n* = 103). Other forms of thyroiditis were Hashimoto's thyroiditis with colloid goiter (*n* = 5), De Quervain's thyroiditis (*n* = 3), and one case each of postpartum thyroiditis and Hashimoto's thyroiditis with associated malignancy. The majority of patients were in the age group of 21–40 (*n* = 70) and the majority (*n* = 73) had diffuse enlargement of thyroid. The majority of patients were hypothyroid (*n* = 52). The serum anti-TPO antibodies were elevated in 47 patients out of 71 patients. In the 48 patients who underwent ultrasonography, 38 were diagnosed as having thyroiditis. The most consistent cytomorphological features seen in fine-needle aspiration smears of Hashimoto's thyroiditis were increased background lymphocytes, lymphocytic infiltration of thyroid follicular cell clusters, and Hurthle cells.

**Conclusion:**

The diagnostic cytological features in Hashimoto's thyroiditis are increased background lymphocytes, lymphocytic infiltration of thyroid follicular cell clusters, and Hurthle cells. FNAC remains the “Gold Standard” for diagnosing Hashimoto's thyroiditis. Clinical history, thyroid function, and biochemical parameters are the key for diagnosis of other forms of thyroiditis.

## 1. Introduction

The prevalence of these thyroid disorders varies widely according to geographical distribution, diet and nutrition, and patient population [1, 2]. Thyroiditis a diverse group of disorder characterized by thyroid inflammation and have different etiologies [3]. It can be categorized as acute, subacute or chronic forms. The most common form of thyroiditis is chronic lymphocytic thyroiditis (Hashimoto's thyroiditis [HT]) [4–9]. Other common types include postpartum thyroiditis (subacute lymphocytic thyroiditis), silent sporadic thyroiditis, subacute granulomatous thyroiditis (De Quervain's thyroiditis), suppurative thyroiditis, and fibrous thyroiditis (Riedel's thyroiditis) [3].

Thyroiditis patients may be euthyroid, hyperthyroid, or hypothyroid and may evolve over the time [10]. Chronic high iodine intake has been associated in various studies with increased frequency of autoimmune thyroiditis [11]. It is well-known that HT can coexist with other lesions such as follicular neoplasm, Hurthle cell neoplasm, papillary carcinoma, and goitrous nodules [12–15]. Incidence of coexisting thyroid neoplasia with HT range between 3 and 14%. Coexistence of thyroiditis and colloid goiter is also seen in significant number of cases [12, 14, 16].

Different types of thyroiditis may share same clinical and biochemical features. Conventional ultrasonography is the most commonly applied modality in the evaluation of thyroiditis [17]. Fine-needle aspiration cytology is practiced worldwide and is the investigation of choice in thyroid enlargement [18–21]. This study has been undertaken to find out the frequency of various thyroiditis and study the cytomorphological features and correlate with clinical findings including radiological features, thyroid function test, and anti-thyroid peroxidase antibodies (Anti-TPO antibodies).

## 2. Materials and Methods

A prospective cohort study was carried out. The study includes consecutive 110 cases of thyroiditis. Inclusion criteria were thyroiditis patients diagnosed on fine-needle aspiration and or ultrasonography, thyroid function tests, and or anti-TPO antibodies. Exclusion criteria were noninflammatory and neoplastic lesions of thyroid.

All the patients underwent fine-needle aspiration (FNA) in cytology clinic. Aspiration/nonaspiration techniques were used. After prior written consent, FNA was done with standard technique and aseptic precautions by using 10cc disposable syringe and 23–25-gauge needle. Aspiration was done from more than one site. Material obtained was smeared on glass slides and smears were stained with Leishman's and Hematoxylin and Eosin (H and E) stains. Detailed cytomorphological features were studied.

Detailed clinical history, ultrasonography findings, serum T3, serum T4, and thyroid stimulating hormone (TSH) and anti-TPO antibodies were noted and correlated with FNA features. The surgical specimens of thyroid were received for histological examination and were formalin-fixed and paraffin-processed. The 3-4 thick micron sections were cut and stained with Hematoxylin and Eosin (H & E).

## 3. Results

Out of 110 thyroiditis patients, majority (*n* = 103) were females and the remaining seven were males. The majority of patients (*n* = 37) were in the age group of 21–30 years followed by 33 patients in the age group of 31–40 years, 17 in 41–50, and 12 in 11–20 and six patients in 61–70 years. Four patients were in between the ages of 51 and 60 years and one patient was between 1 and 10 years. The majority of the patients in our study were diagnosed as HT (*n* = 100), followed by HT with colloid goiter (*n* = 5) and De Quervain's thyroiditis (*n* = 3) and one case each of postpartum thyroiditis and HT with associated malignancy (Table 1).

All the patients presented with goiter. The 73 patients presented with diffuse enlargement of thyroid, 34 patients with uneven enlargement, and 03 patients with solitary nodule. Table 1 shows detailed correlation of nature of goiter, thyroid function test, and anti-TPO antibodies in 110 patients of thyroiditis.

Ultrasonography findings of thyroid were available in 48 patients. The 38 patients were diagnosed as thyroiditis and showed features such as diffusely altered parenchyma with heterogeneous echogenicity, hypovascular goiter, micronodules, ill-defined hypoechogenic regions, and marked fibrosis. One case showed dominant nodule which turned out to be papillary carcinoma of thyroid in HT on FNA and histopathological examination. The 10 patients were diagnosed as multinodular goiter.

FNA cytology smears in all cases showed increased background lymphocytes. The lymphocytic infiltration of thyroid follicular cell clusters was seen in 79 cases and Hurthle cells in 64 cases. Other associated features such as mild anisonucleosis, giant cells, histiocytes, scanty colloid, epithelioid cells, plasma cells, fire flares, and eosinophils were seen in few cases.  Table 2 shows the frequency of cytomorphological features in 100 cases of HT. Based on severity of lymphocytic infiltrate and presence or absence of Hurthle cells in cytology smears, we categorized HT cases into three groups (Table 3).

We came across three patients of De Quervain's thyroiditis in our study. All three patients presented with painful uneven enlargement of thyroid; thyroid function was variable and, in all patients, anti-TPO titers were normal (Table 1). FNA smears in all cases showed many large multinucleate giant cells, epithelioid cells, and lymphocytes (Figures 2(a) and 2(b)). Clinical history and other laboratory investigations such as elevated acute phase reactants established the diagnosis.

Out of five cases of HT with colloid goiter, the majority of patients presented with uneven thyroid enlargement (*n* = 4), three patients were hypothyroid, and in two cases anti-TPO titers were elevated (Table 1). FNA smears in all cases showed colloid on the background and evidence of thyroiditis (Figure 3(a)). In three cases partial thyroidectomy was done due to pressure symptoms and diagnosis was confirmed by histopathological examination (Figure 3(b)).

The only case of postpartum thyroiditis in our study was a 27-year-old woman who had delivered a child 7 months back. She presented with painless goiter. FNA smears showed lymphocytic thyroiditis. Anti-TPO titer was not elevated (Table 1).

The only case of HT with carcinoma showed evidence of increased lymphocytes on background, occasional thyroid follicular cell cluster infiltrated by lymphocytes,and many scattered large atypical cells having large nuclei and intranuclear inclusion (Figures 4(a) and 4(b) arrow).

Anti-TPO antibody titer was raised. Surgical specimen received for histological examination revealed HT with papillary thyroid carcinoma (PTC) (Figure 4(c)).

## 4. Discussion

Thyroiditis encompasses many relatively common thyroid disorders which have been classified into (1) HT (Chronic lymphocytic thyroiditis), (2) postpartum thyroiditis, (3) painless sporadic thyroiditis, (4) De Quervain's thyroiditis (Subacute granulomatous thyroiditis), (5) suppurative thyroiditis, (6) drug-induced thyroiditis, and (7) Riedel's thyroiditis [3]. The introduction of FNA 40 years back has substantially improved the preoperative assessment of thyroid lesions due to its high positive and negative predictive value [22–24]. HT and De Quervain's thyroiditis are the most commonly encountered although overlap of both these conditions is documented [25–27]. In our study the majority of patients were of HT (*n* = 100).

The male-to-female ratio in our study was 1 : 14.7. Thyroiditis was more common in females (*n* = 103) than males (*n* = 7). The majority of our patients (*n* = 100) had HT. Maximum number of patients (*n* = 68) were in between the ages of 21 and 40 years. Our results were in accordance with many studies [6, 7, 9, 12, 28–31]. In contrast, in Italian study, male-to-female ratio was only 1 : 3.2 [32]. The only case of De Quervain's thyroiditis in our study was a 45-year-old female.

Similar age group was observed by Nishihara et al. [33]. In contrast Vanderpump et al. [34] found a man aged 59 years with HT at the time of diagnosis. A greater number of younger patients in our study can be explained by the fact that the area they live in is a noncostal area which can be iodine-deficient. In these areas, HT is known to occur in young patients [26]. Other reason can be early diagnosis.

The majority of patients in our study (*n* = 73) had diffuse enlargement of thyroid. Discrete nodular presentation is well known in HT [12]. Higher number of discrete nodularities (*n* = 34) in our study can be due to younger age of the patients and early stage of the disease at the time of diagnosis. Out of three solitary nodules, one case was eventually turned out to be HT with papillary carcinoma of thyroid.

Thyroid function tests were done in all patients. Large number of hypothyroid patients (*n* = 52) in our study can be explained by the fact that majority of thyroiditis patients had HT and possibly the advanced stage of the disease at the time of diagnosis. Substantial number of patients had hyperthyroidism (*n* = 27). This can be explained by the fact that hashitoxicosis is a transient hyperthyroid phase of HT.

Anti-TPO antibody titers were done in 71 cases out of which 47 patients had elevated titers. Autoantibodies against thyroglobulin and thyroid peroxidase antigens are clinically most important for diagnosis. Elevated titers have been in 95% of the patients [6]. There is a controversy whether anti-TPO alone is sufficiently reliable to diagnose HT. Anti-TPO negative cases are known to occur. Up to 20% adult females with no clinical disease have detectable Tg/TPO antibodies, raising the question about their pathologic significance.

**Figure 1:**
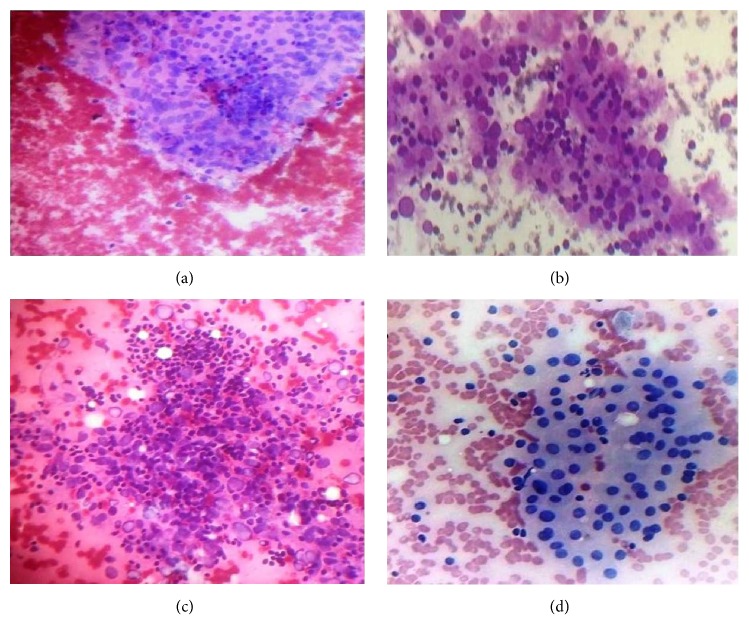
Hashimoto's thyroiditis: (a) Group 1 smears showing follicular cell clusters infiltrated by few lymphocytes (Leishman's stain ×100); (b) Group 2 smears showing follicular cell clusters infiltrated by moderate amount of lymphocytes (Leishman's stain ×400); (c) Hurthle cells and increased background lymphocytes (Leishman's stain ×400); (d) dense lymphocytes in follicular cells clusters (Haematoxylin & Eosin ×100).

**Figure 2:**
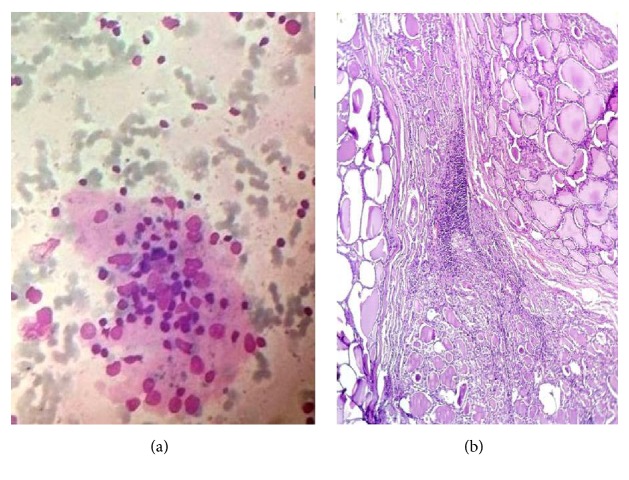
Hashimoto's thyroiditis with colloid goiter: smears showing thin background colloid and follicular cell cluster infiltrated by lymphocytes (Leishman's stain ×400); (b) Hashimoto's thyroiditis with colloid goiter (Haematoxylin & Eosin ×400).

Thyroid imaging is nowadays an essential part of thyroid disease evaluation and it may aid in establishing proper diagnosis. The most commonly applied modality in evaluation of thyroiditis is conventional ultrasonography [17]. Similar observations were made in our study. The 10 patients diagnosed as MNG turned out as HT on FNA. The most consistent cytological features seen in FNA smears of HT were increased background lymphocytes, lymphocytic infiltration of thyroid follicular cell clusters, and Hurthle cells. HT shows two patterns in cytology smears which correspond to different phases of the disease: (1) classic HT, in which smears show increased background lymphocytes and infiltration of follicular cell clusters by lymphocytes (Figures 1(a), 1(b), and 1(c)); (2) florid lymphocytic pattern in which smears show dominant lymphoid cell population in stages of maturation. Epithelial cells may be inconspicuous (Figure 1(d)). Similar observations were made in our study. Florid lymphocytic thyroiditis at times can be difficult to differentiate from lymphoma in cytology smears especially in adults. But careful search for other cytological features of HT reduces the diagnostic errors. It is now widely accepted that florid lymphocytic thyroiditis and HT represent different manifestations of autoimmune thyroiditis.

HT patients were grouped into Group 1, Group 2, and Group 3 based on severity of lymphocytic infiltrate and were correlated with thyroid function and anti-TPO titers. Group 1, Group 2, and Group 3 suggested milder early stage, more severe form and late stage of the disease, respectively. Other cytomorphological features listed in Table 2 were seen in variable numbers in FNA smears.

Scanty colloid is not an usual feature in FNA smears of HT (*n* = 8). But careful search for lymphocytic infiltrate and repeat aspiration from other area establish the diagnosis of HT. Other associated features such as mild anisonucleosis (*n* = 19), few giant cells (*n* = 19), and histiocytes (*n* = 12), though were not diagnostic, were seen in variable numbers in cytology smears and this is known to occur. Fire flares were noted in few patients of HT (*n* = 3). Similar frequency was noted by Rathi et al. [29]. In contrast, some studies noted slightly more frequency [26, 35]. It is known that fire flares can be seen in variety of benign and malignant thyroid lesions. Ekambaram et al. [36] found eosinophils in 84% patients of HT in thyroid smears. In contrast, we found eosinophils in only one cases.

Other autoimmune disorders may coexist with HT [37]. We did not find any significant evidence of any other autoimmune disorders in any patients with the available history.

**Figure 3:**
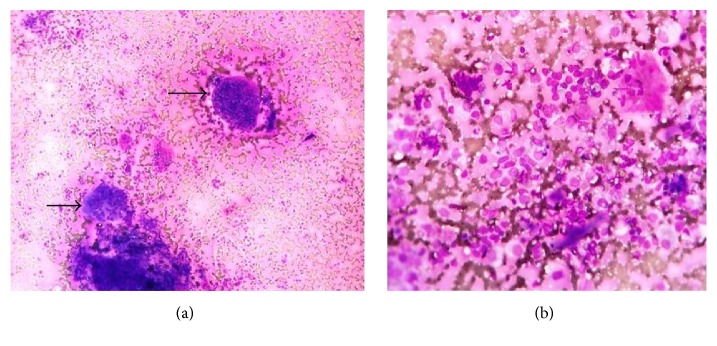
De Quervain's thyroiditis: (a) smears showing follicular cell clusters with many giant cells (thick arrow, Leishman's stain ×100); (b) epithelioid cells and lymphocytes (Leishman's stain ×400).

**Figure 4:**
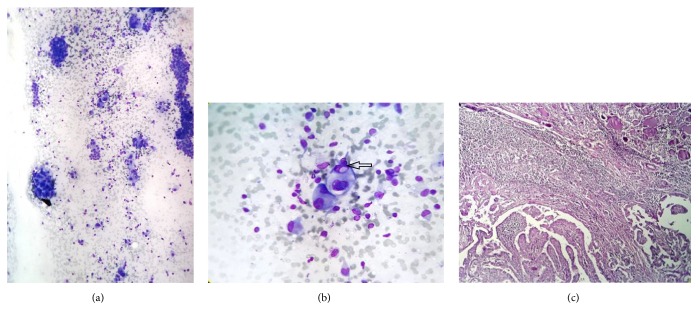
Hashimoto's thyroiditis with thyroid carcinoma: (a) smears showing many large atypical cells (Leishman's stain ×400); (b) atypical follicular cells with intranuclear cytoplasmic inclusion; (c) tissue section showing papillary thyroid carcinoma (Haematoxylin & Eosin ×400).

FNA diagnosis of HT with colloid goiter was made in few cases (*n* = 5). Clinical findings and radiological findings suggested colloid goiter. FNA smears in these patients showed moderate to severe background colloid and lymphocytes infiltrating follicular cell clusters and Hurthle cells (Figure 3(a)). Anti-TPO titers were done in all five cases out of which titers were elevated in two cases (Table 2). Surgical specimens of partial thyroidectomy were received in three cases for histopathological examination and confirmed the diagnosis of colloid goiter with HT (Figure 3(b)).

Neoplastic and nonneoplastic lesions are known to be associated with HT like colloid goiter, follicular adenoma, Hurthle cell neoplasm, papillary thyroid carcinoma (PTC), non-Hodgkin's lymphoma (NHL), and follicular carcinoma [38]. In various surgical series, the prevalence of malignancy in HT ranges from 0.4% to 28% [39, 40]. The most commonly encountered neoplasms in association with HT are papillary thyroid carcinoma and primary thyroid lymphoma [41]. Similar observations were made in our study. We came across only one case of HT with PTC in our study. We missed definitive diagnosis of HT with PTC because neoplastic component was scanty and did not show definitive features of PTC.

We came across three patients of De Quervain's thyroiditis. They were between the ages of 31 and 50 years. All the three patients complained of painful goiter. FNA features, painful goiter, and elevated erythrocyte sedimentation rate and C-reactive protein established the definitive diagnosis. Etiology is probably viral. In majority of the cases, there is compete resolution [42].

Postpartum thyroiditis is a variant of autoimmune thyroiditis. It occurs during the postpartum period in up to 5% of women. The 1/3 of cases can evolve into overt hypothyroidism over a period of 10 years. Most of the patients have elevated circulating anti-TPO. Similar observation was made in our study (Table 1). We conclude that HT is the most common form of thyroiditis and it occurs most commonly in young and middle age women. The diagnostic cytological features seen in FNA smears of HT are increased background lymphocytes, lymphocytic infiltration of thyroid follicular cell clusters, and Hurthle cells. Clinical history, anti-TPO, thyroid function test, and ultrasonography are very important and useful adjuncts in the diagnosis of HT. FNAC remains the “Gold Standard” for diagnosing Hashimoto's thyroiditis. Clinical history, thyroid function, and biochemical parameters are key for diagnosis of other forms of thyroiditis.

## Figures and Tables

**Table 1:** Correlation of goiter, thyroid function, and anti-TPO in 110 cases of thyroiditis.

Type of thyroiditis (*n* = number)	Goiter			Thyroid function				Anti-TPO titer Number of cases/increased titer
	Diffuse	Uneven	SN	Hypo	Hyper	SCH	Euth	
Hashimoto thyroiditis (*n* = 100)	71	27	02	47	25	15	13	61/44
HT with colloid goiter (*n* = 5)	01	04	00	03	00	00	02	5/2
de Quervain thyroiditis (*n* = 3)	00	03	00	01	01	00	01	3/0
Postpartum thyroiditis (*n* = 1)	01	00	00	00	01	00	00	1/0
HT with malignancy (*n* = 1)	00	00	01	01	00	00	00	1/1

Total-110	73	34	03	52	27	15	16	71/47

HT: Hashimoto's thyroiditis; SN: solitary nodule; Hyper: hyperthyroidism; Hypo: hypothyroidism; SCH: subclinical hypothyroidism; Euth: euthyroidism.

**Table 2:** Frequency of various cytological features in Hashimoto's thyroiditis.

Cytomorphological features	Number of cases	Percentage (%)
Increased background lymphocytes	100	100
Lymphocytic infiltration of follicular cell clusters	79	79
Hurthle cells	64	64
Mild anisonucleosis	19	19
Few giant cells	18	18
Histiocytes	12	12
Scanty colloid	8	8
Epithelioid cells	6	6
Plasma cells	5	5
Fire flares	3	3
Eosinophils	1	1

**Table 3:** Cytomorphological features of three groups of HT patients.

Groups	Number of cases	Cytomorphological features
1	46	Mild lymphocytic infiltrate in follicular cells with or without Hurthle cells
2	40	Moderate lymphocytes, follicular cell destruction, and Hurthle cells
3	14	Dense lymphocytes/lymphoid cells in various stages of transformation with very few follicular and Hurthle cells

## References

[1] Shukla S., Acharya S., Pansey P., Dawande P., Tamhane A. (2015). Epidemiology of thyroid lesions in Wardha District of Central India. *Journal of Evidence Based Medicine and Healthcare*.

[2] Nagarkar R., Roy S., Akheel M., Palwe V., Kulkarni N., Pandit P. (2015). Incidence of thyroid disorders in India: An institutional retrospective analysis. *International Journal of Dental and Medical Specialty*.

[3] Pearce E. N., Farwell A. P., Braverman L. E. (2003). Thyroiditis. *The New England Journal of Medicine*.

[4] Vanderpump M. P., Tunbridge W. M. (2009). Epidemiology and prevention of clinical and subclinical hypothyroidism. *Thyroid Int*.

[5] Mönig H., Harbeck B. (2008). Thyroiditis. *Deutsche Medizinische Wochenschrift*.

[6] Anila K., Nayak N., Jayasree K. (2016). Cytomorphologic spectrum of lymphocytic thyroiditis and correlation between cytological grading and biochemical parameters. *Journal of Cytology*.

[7] Bhatia A., Rajwanshi A., Dash R. J., Mittal B. R., Saxena A. K. (2007). Lymphocytic Thyroiditis - Is cytological grading significant? A correlation of grades with clinical, biochemical, ltrasonographic and radionuclide parameters. *CytoJournal*.

[8] Chandanwale S., Gore C., Bamanikar S., Gupta N., Gupta K. (2014). Cytomorphologic spectrum of Hashimoto′s thyroiditis and its clinical correlation: A retrospective study of 52 patients. *CytoJournal*.

[9] Kamra H. T., Agarwal R., Rana P. (2014). Evaluation profile of thyroid nodule by fnac in the rural population of Khanpur Kalan, Sonepat, Haryana. *Journal of Clinical and Diagnostic Research*.

[10] Sen R., Gupta M., Sachdeva B. (2006). Thyroid—An update of diagnostic pathology. *Journal of Postgraduate Medical Education, Training & Research*.

[11] Duntas L. H. (2015). The Role of Iodine and Selenium in Autoimmune Thyroiditis. *Hormone and Metabolic Research*.

[12] Nguyen G.-K., Ginsberg J., Crockford P. M., Villanueva R. R. (1997). Hashimoto's thyroiditis: Cytodiagnostic accuracy and pitfalls. *Diagnostic Cytopathology*.

[13] Zeppa P., Benincasa G., Lucariello A., Palombini L. (2001). Association of different pathologic processes of the thyroid gland in fine needle aspiration samples. *Acta Cytologica*.

[14] Carson H. J., Castelli M. J., Gattuso P. (1996). Incidence of neoplasia in Hashimoto's thyroiditis: A fine-needle aspiration study. *Diagnostic Cytopathology*.

[15] Kebebew E., Treseler P. A., Ituarte P. H. G., Clark O. H. (2001). Coexisting chronic lymphocytic thyroiditis and papillary thyroid cancer revisited. *World Journal of Surgery*.

[16] Kumarasinghe M. P., De Silva S. (1999). Pitfalls in cytological diagnosis of autoimmune thyroiditis. *Pathology*.

[17] Ruchala M., Szczepanek-Parulska E. (2013). Imaging techniques in diagnostics, differentiation and monitoring of different types of thyroiditis. *Thyroid Research*.

[18] De Micco C., Zoro P., Garcia S. (1994). Thyroid peroxidase immunodetection as a tool to assist diagnosis of thyroid nodules on fine-needle aspiration biopsy. *European Journal of Endocrinology*.

[19] Tabaqchali M. A., Hanson J. M., Johnson S. J., Wadehra V., Lennard T. W., Proud G. (2000). Thyroid aspiration cytology in Newcastle: A six year cytology/histology correlation study. *Annals of The Royal College of Surgeons of England*.

[20] Nggada H., Khalil M. (2003). Fine needle aspiration cytology [FNAC] technique as a diagnostic tool of tumours in the University of Maiduguri Teaching Hospital, Nigeria. *Highland Medical Research Journal*.

[21] Kini U., Buch A., Bantwal G. (2006). Role of FNA in the medical management of minimally enlarged thyroid. *Diagnostic Cytopathology*.

[22] Raza S., Saeed Z., Raza H., Ahmed M. (2006). FNAC in management of solitary thyroid nodule. *Professional Medical Journal*.

[23] Handa U., Garg S., Mohan H., Nagarkar N. (2008). Role of fine needle aspiration cytology in diagnosis and management of thyroid lesions: a study on 434 patients. *Journal of Cytology*.

[24] Gupta M., Gupta S., Gupta V. B. (2010). Correlation of fine needle aspiration cytology with histopathology in the diagnosis of solitary thyroid nodule. *Journal of Thyroid Research*.

[25] Clark D. P., Faquin W. C., Rosenthal D. L. (2010). Inflammatory lesions and lymphoma. *Thyroid Cytopathology*.

[26] Jayaram G., Marwaha R. K., Gupta R. K., Sharma S. K. (1987). Cytomorphologic aspects of thyroiditis. A study of 51 cases with functional, immunologic and ultrasonographic data. *Acta Cytologica*.

[27] Kumar N., Ray C., Jain S. (2002). Aspiration cytology of Hashimoto's thyroiditis in an endemic area. *Cytopathology*.

[28] Sood N., Nigam J. S. (2014). Correlation of fine needle aspiration cytology findings with thyroid function test in cases of lymphocytic thyroiditis. *Journal of Thyroid Research*.

[29] Rathi M., Ahmad F., Budania S. K., Awasthi S., Kumar A., Dutta S. (2014). Cytomorphological aspects of Hashimoto's thyroiditis: Our experience at a tertiary center. *Clinical Medicine Insights: Pathology*.

[30] Kalita A., Baruah R. (2015). Thyroiditis: a Clinico-cytomorphological Study with a Reference to the Ethnic Groups of Northeast Regions of India. *Indian Journal of Otolaryngology and Head & Neck Surgery*.

[31] Siriweera E. H., Ratnatunga N. V. I. (2010). Profile of Hashimoto's thyroiditis in Sri Lankans: Is there an increased risk of ancillary pathologies in Hashimoto's thyroiditis?. *Journal of Thyroid Research*.

[32] Martino E., Buratti L., Bartalena L. (1987). High prevalence of subacute thyroiditis during summer season in Italy. *Journal of Endocrinological Investigation*.

[33] Nishihara E., Ohye H., Amino N. (2008). Clinical characteristics of 852 patients with subacute thyroiditis before treatment. *Internal Medicine*.

[34] Vanderpump M. P. J., Tunbridge W. M. G., French J. M. (1995). The incidence of thyroid disorders in the community: a twenty-year follow-up of the Whickham Survey. *Clinical Endocrinology*.

[35] Jayaram G., Iyengar K., Sthaneshwar P., Hayati J. (2007). Hashimoto’s thyroiditis—A Malaysian Perspective. *Journal of Cytology*.

[36] Ekambaram M., Kumar B., Chowdhary N., Siddaraju N., Kumar S. (2010). Significance of eosinophils in diagnosing Hashimoto's thyroiditis on fine-needle aspiration cytology. *Indian Journal of Pathology and Microbiology*.

[37] Gulec M., Kartal O., Caliskaner A. Z. (2011). Chronic urticaria in patients with autoimmune thyroiditis: Significance of severity of thyroid gland inflammation. *Indian Journal of Dermatology, Venereology and Leprology*.

[38] Singh N., Kumar S., Negi V. S., Siddaraju N. (2009). Cytomorphologic study of Hashimoto's thyroiditis and its serologic correlation: A study of 150 cases. *Acta Cytologica*.

[39] Shih M.-L., Lee J. A., Hsieh C.-B. (2008). Thyroidectomy for Hashimoto's thyroiditis: Complications and associated cancers. *Thyroid*.

[40] Matesa-Anic D., Matesa N., Dabelic N., Kusic Z. (2009). Coexistence of papillary carcinoma and Hashimotos thyroiditis. *Acta Clinica Croatica*.

[41] Singh B., Shaha A. R., Trivedi H., Carew J. F., Poluri A., Shah J. P. (1999). Coexistent Hashimoto's thyroiditis with papillary thyroid carcinoma: impact on presentation, management, and outcome. *Surgery*.

[42] Maitra A., Kumar V., Abbas A. K., Aster J. C. (2014). The Endocrine System. *Robbins & Cotran Pathologic Basis of Disease*.

